# Identification of distant co-evolving residues in antigen 85C from *Mycobacterium tuberculosis* using statistical coupling analysis of the esterase family proteins

**DOI:** 10.1016/S1674-8301(11)60021-3

**Published:** 2011-05

**Authors:** Veeky Baths, Utpal Roy

**Affiliations:** Department of Biological Sciences, BITS Pilani K. K Birla Goa Campus, GOA 403726, India.

**Keywords:** antigen 85C, *Mycobacterium tuberculosis*, clustering analysis, covariance, statistical coupling analysis, esterase family, multiple sequence alignments, pfam, Protein Data Bank.

## Abstract

A fundamental goal in cellular signaling is to understand allosteric communication, the process by which signals originating at one site in a protein propagate reliably to affect distant functional sites. The general principles of protein structure that underlie this process remain unknown. Statistical coupling analysis (SCA) is a statistical technique that uses evolutionary data of a protein family to measure correlation between distant functional sites and suggests allosteric communication. In proteins, very distant and small interactions between collections of amino acids provide the communication which can be important for signaling process. In this paper, we present the SCA of protein alignment of the esterase family (pfam ID: PF00756) containing the sequence of antigen 85C secreted by *Mycobacterium tuberculosis* to identify a subset of interacting residues. Clustering analysis of the pairwise correlation highlighted seven important residue positions in the esterase family alignments. These residues were then mapped on the crystal structure of antigen 85C (PDB ID: 1DQZ). The mapping revealed correlation between 3 distant residues (Asp38, Leu123 and Met125) and suggests allosteric communication between them. This information can be used for a new drug against this fatal disease.

## INTRODUCTION

Communication between distant sites in proteins is fundamental to their function and often defines the biological role of a protein family. In signaling proteins, it represents information transfer -- the transmission of signals initiated at one functional surface to a distinct surface mediating downstream signaling. For example, ligand binding at an externally accessible site in G protein-coupled receptors (GPCRs) reliably triggers structural changes at distant cytoplasmic domains that mediate interaction with heterotrimeric G proteins[1],[2]. Studies in many other protein systems indicate that long-range interactions of amino acids also are important in binding (and catalytic) specificity. Substrate recognition in the chymotrypsin family of serine proteases[3],[4], the tuning of antibody specificity through B-cell maturation[5] and the cooperativity of oxygen binding in hemoglobin[6] all depend not only on residues directly contacting the substrate, but also on distant residues located in supporting loops and other secondary structural elements.

Statistical coupling analysis (SCA) is a technique used to identify communication between distant sites in proteins. More specifically, it quantifies how much the amino acid distribution at some position *i* changes upon a perturbation of the amino acid distribution at another position *j*. The resulting statistical coupling energy indicates the degree of evolutionary dependence between the residues, with higher coupling energy corresponding to increased dependence. Previous application of SCA done on the PDZ domain family [(*PDZ* is an acronym combining the first letters of three proteins — post synaptic density protein (PSD95), *Drosophila* disc large tumor suppressor (DlgA), and zonula occludens-1 protein (zo-1)]. These PDZ domain family proteins were first discovered to share the domain[7]. Apart from sharing of domains, this protein family has also got a predicted set of energetically coupled positions for the binding site residue that contained unexpected long-range interactions. Further mutational analysis confirmed the prediction that statistical energy function is a good indicator for thermodynamic coupling in proteins[8]. Application of SCA to three structurally and functionally distinct protein families (GPCR, the chymotrypsin class of serine proteases and hemoglobin) revealed a simple architecture for amino acid interaction in protein families and link distant functional sites in the tertiary structure[9]. Further application to the S1A serine protease family indicated the presence of quasi-independent groups of correlated amino acids termed as “Protein Sectors”. Each of these sectors was found to be physically connected in the tertiary structure, had a distinct functional role and constituted an independent mode of sequence divergence in the protein family[10].

The antigen 85 (ag85) complex, composed of three proteins (ag85 A, B and C), is a major component of the *Mycobacterium (M.) tuberculosis* cell wall. Each protein possesses a mycolyltransferase activity required for the biogenesis of trehalose dimycolate (cord factor), a dominant structure necessary for maintaining cell wall integrity[11],[12]. The protein sequence of ag85 C, which is a member of the esterase family proteins, seems to be responsible for the high affinity of *Mycobacterium* to fibronectin[12]. Since ag85 proteins are important for cell wall biosynthesis, it could be a target for a novel antitubercular drug. With the aim of identifying residue positions, which could be responsible for the function of ag85 C and hence act as potential drug targets for *M. tuberculosis*, we applied SCA to the esterase protein family.

## MATERIALS AND METHODS

### Methods

**Fig. 1:**
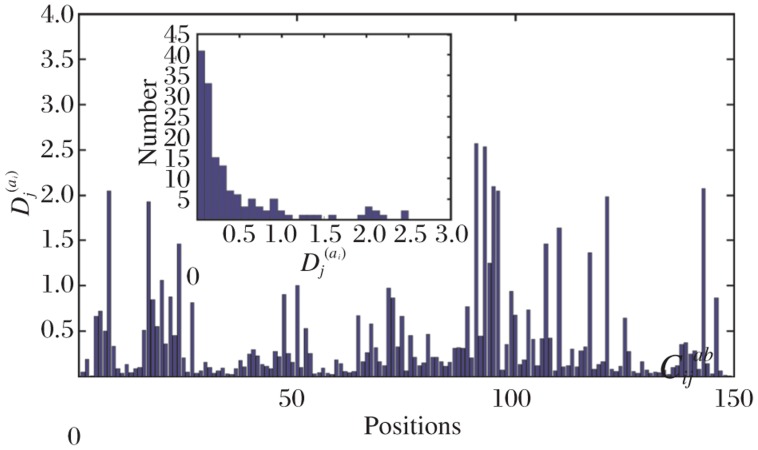
Sequence conservation in the esterase family multiple sequence alignment. The degree of sequence conservation is plotted along the protein sequence with high values indicating high conservation. The plot corresponds to the diagonal elements.

The SCA was performed as described by Halabi *et al*.[10]. Briefly (details in Results), each site i in the multiple sequence alignment (MSA) was assigned a sequence conservation parameter *D_i_*^(*a*)^. As an example, the *D_i_*^(*a*)^ scores for one of the test proteins were plotted in ***Fig. 1***. The correlated mutation score *C_ij_^ab^* expresses difference between the *D_i_*^(*a*)^ and in the perturbed alignment where the amino acid *j* was constrained. Instead of choosing particular residue positions, which constrain the MSA, a series of perturbations were carried out by sequentially eliminating a sequence at a time from the alignment. The fluctuation of sequence conservation at each site is recorded to give perturbation trajectories for each amino acid at each site. Sites which are not evolutionarily coupled are expected to show independent patterns of fluctuation, while sites that are coupled are expected to show some mutual dependence in their fluctuations. The final output is a matrix of sequence alignment length contacting scalar coupling value for each pair of positions.

### Datasets

The putative esterase family (pfam ID: PF00756 alignment (of 4945 sequences) was downloaded (updated till April 2010) from the pfam database (http://pfam.sanger.ac.uk/)[10]. Sequence redundancies were removed from the database at the threshold value of 99% using JalView[14] (846 out of 4945 sequences left). The dataset was realigned using ClustalX and no further manual adjustments were done[15]. The dataset was further refined by the removal of all columns with more than 20% gaps (using MATLAB) (149 columns left). This prevented any trivial over-representation of gaps in the alignment and ensured that the calculations were only made at largely non-gapped sequences positions. SCA was performed using the code provided by Ranganathan[10]. Clustering analysis was performed in the form of hierarchical clustering analysis by construction of dendrograms of the correlation matrix using MATLAB. Coupled residues were mapped on to the structure of the secreted form of ag85 C and the crystal structure of the secreted form of ag85 C (PDB ID: 1DQZ) was available on the PDB database for structural analysis.

## RESULTS

The conservation of an amino acid *a* at position *i* in a multiple sequence alignment is defined by *D_i_*^(*a*)^ the divergence (or relative entropy) of the observed frequency of *a* at *I* (*fi*^(*a*)^) from the background frequency of *a* in all proteins (*q*^(*a*)^)[8]. 
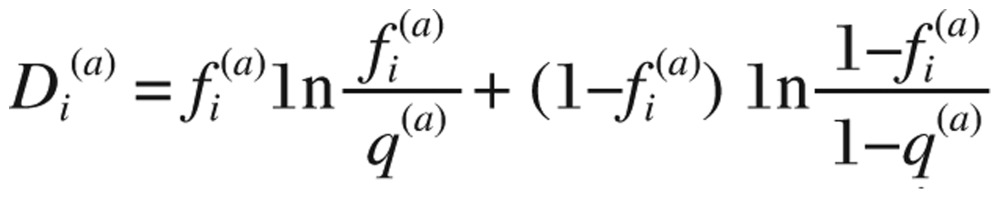

*D_i_*^(*a*)^ is a non-linear function of *f_i_*^(*a*)^ that rises more and more steeply as *f_i_*^(*a*)^ approaches one. As a practical consequence, for all but the least conserved positions, the overall conservation of all amino acids at each position *i* is well approximated by *D_i_*^(*ai*)^, the conservation of *a_i_*, the most prevalent amino acid at that position[13].

***Fig. 1*** shows the positional conservation of all the 149 residue positions (left after removal of redundancy and gap reduction on the original downloaded MSA. We call the new alignment: “truncated alignment”) in the truncated alignment of the esterase family. It also shows the distribution of *D_i_*^(*a*)^ values of all the positions as a measure of conservation.

**Fig. 2:**
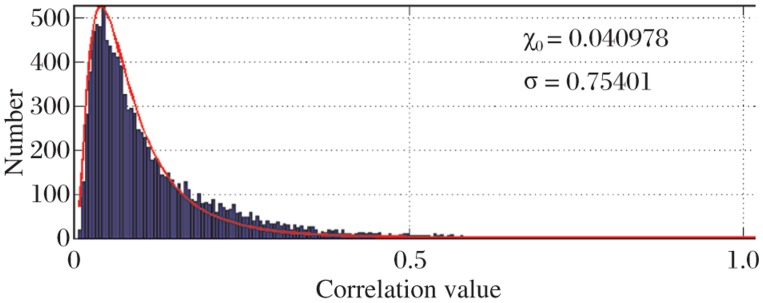
Histogram of pairwise correlation values. The figure suggests that only a few pairs correlate with a significant correlation where as most of the correlation values (99%) lie around the mean value of 0.040978.

Histogram of pairwise correlation values of the 149 residue positions as shown in ***Fig. 1*** indicates that most of the values (nearly 99%) lie close to the mean value of the histogram and only a few pairs show significant correlation and only about 1% of the total pairs show significant correlation (values greater than e^Xo + 2.36σ^). This indicates that only a few pairs of residues correlate with each other with high correlation values. This essentially explains that in putative esterase protein family only a few residue pairs show interaction and communicate with each other even from a long range. According to the CASP guidelines (http://www.predictioncenter.llnl-.gov/CASP9), a “long-range” inter-residue contact is defined as two residues that are separated by at least nine residue positions in the linear sequence in which Cβ-Cβ distance is ≤ 8 Å[16]. ***Fig. 2*** shows the correlation values.

**Fig. 3:**
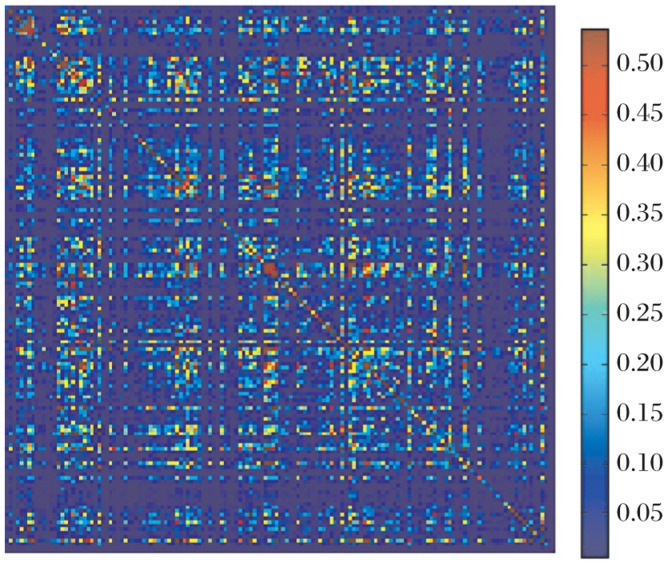
Heat map of the correlation matrix. The red pixels represent high correlation values and as we scale down the blue pixels represent low correlation values.

The result of the correlation is shown in terms of a correlation matrix, which is represented as a heat map (***Fig. 3***). The basic principle of the SCA correlation matrix, is to weigh the frequency-based correlations between positions *i* and *j*, 
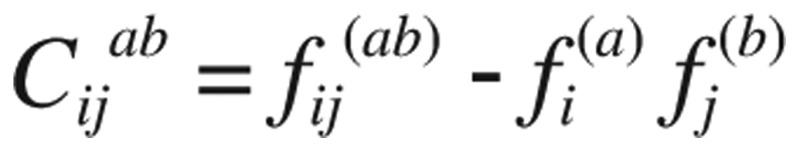
, by a function of their positional conservations which is given by *D_i_*^(*a*)^ and *D_i_* *^(b)^*. 
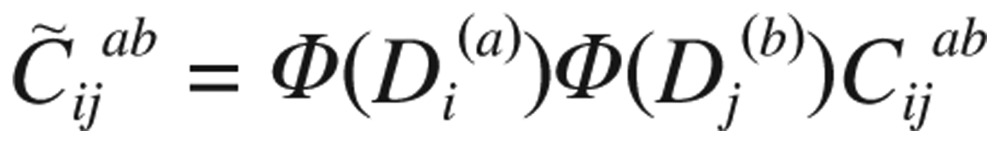


Thus 
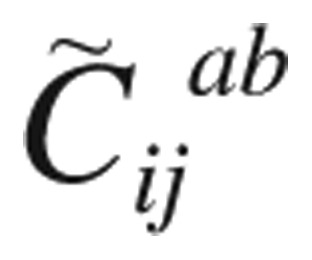
 is a measure of the significance of observed correlations as judged by the conservation of the amino acids under consideration. Following the earlier work, we chose the weighting functions here to be gradients of positional conservation: φ =∂∂*D* / ∂*f* [8]-[10].

**Fig. 4:**
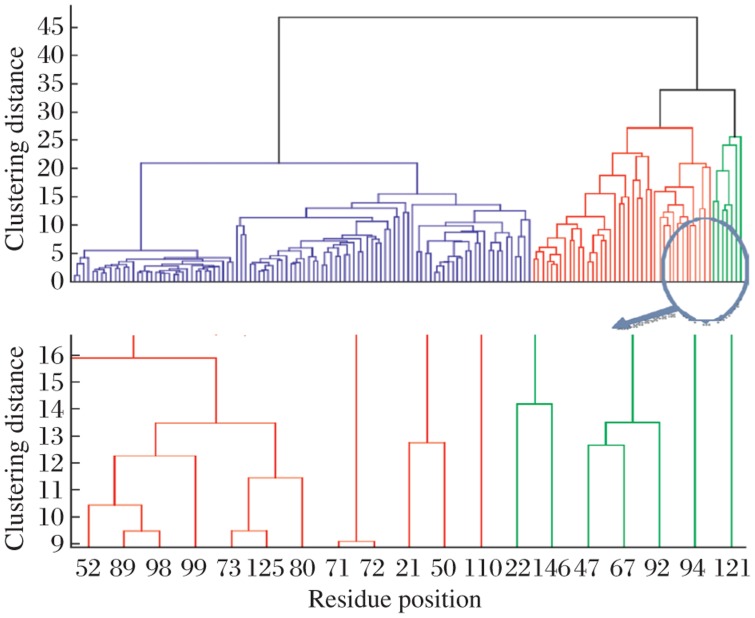
Dendogram representing clustering analysis of pairwise correlation values. The cluster represented in green is the cluster with the most significantly coupled residue positions. X-axis represents the residue position in the truncated alignment and Y-axis represents the clustering distance between any two-residue positions. The arrow indicates a cluster of seven residues.

For a correlation matrix M, the value M(i,j) would mean the correlation value between the ith and jth residue in the multiple sequence alignment (MSA). The heat map in ***Fig. 3*** is a representation of this correlation matrix for the esterase family alignments where most of the pixels are blue to indicate that most of the correlation values are significantly low. However, there are few red spots, highlighting the significant correlations. The diagonal values represent auto-correlation values, which is a representation of the conservation of that particular position. In order to identify the pairs of residues with high correlation values, we performed hierarchical clustering. This is a method of cluster analysis, which seeks to build a hierarchy of clusters. Hierarchical clustering enables grouping of the data over a variety of scales by creating a cluster tree or *dendrogram*. The tree is not a single set of clusters, but rather a multilevel hierarchy, where clusters at one level are joined as clusters to the next level[17]. ***Fig. 4*** represents the result obtained by performing hierarchical clustering on the pair-wise correlation. Values obtained from the SCA of the esterase family alignments as can be seen in ***Fig. 4*** where a cluster of 7 residues (in green) was identified. Positions 22, 47, 67, 92, 94, 121 and 146 from the truncated alignment formed the cluster of closely related residue position, which correlated with a high correlation value. This essentially means that these residue positions are statistically correlated to one other with high correlation value. Our hypothesis is that these are significant to the esterase family protein functions.

**Fig. 5:**
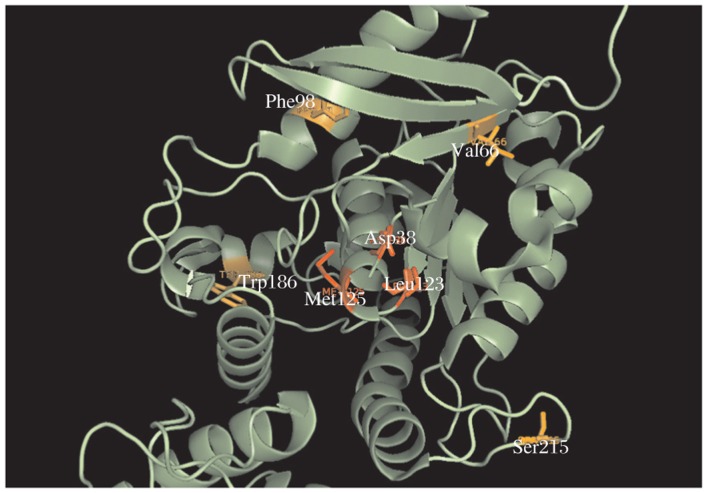
Mapping of the 7 residue positions highlighted by SCA onto the crystal structure of antigen 85 C (PDB ID: 1DQZ). The residues shown are Asp38, Val66, Phe98, Leu123, Met125, Trp186 and Ser215. The pairs in red Asp38, Leu123 and Asp38, Met125 seem to show physical interaction through allostery.

Esterase family alignments contain the sequence of ag85 C secreted by *M. tuberculosis*. We mapped these residue positions from the truncated alignment onto the crystal structure of the secreted form of ag85 C manually. ***Fig. 5*** represents the mapping of seven residues. There are a total of 8-282 residues, the apo form of ag85 C (PDB accession 1DQZ)

**Fig. 6:**
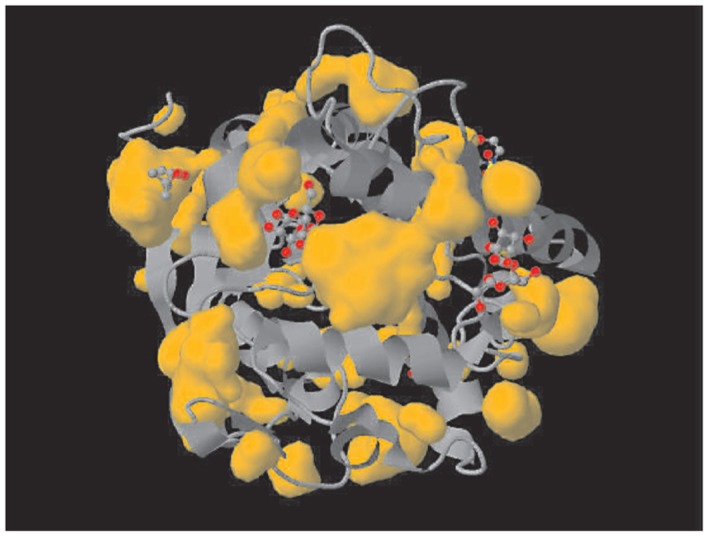
Structures of the *Mycobacterium tuberculosis* 30 kDa major secretory protein (Antigen 85B). A mycolyl transferase (PDB ID: 1FOP), another esterase showing similar pocket.

As can be seen in ***Fig. 5***, the residues that have been mapped are Asp 38, Val 66, Phe 98, Leu 123, Met 125, Trp 186 and Ser 215. A close look at the structural positions of these residues highlights some interesting and important information about these residues and ag85 C. Three of the seven residues (shown in red in ***Fig. 5***) seem to be physically interacting in the form of electrostatic interactions, and further Asp38 and Met125, Asp38 and Leu123 also qualify for the CASP definition of long-range residues[16]. ***Fig. 6*** shows other esterase.

## DISCUSSION

In this work, we have presented the SCA of MSA of putative esterase family of proteins, which is known to contain several seemingly unrelated proteins. Three (Asp38, Leu123 and Met125) out of seven residues as identified by SCA seem to interact physically as seen from the mapping on the crystal structure of ag85 C (***Fig. 5***). The pair Arg 38 and Met 125 has been previously reported to be important for the function of ag85 C. Arg38 is one of the residues, which form a pocket with a high negative electrostatic potential. Ag85 C when complexed with a covalent inhibitor implicates residues Leu40 (close to Arg38) and Met125 as components of oxyanion hole[10]. Mutating such a pair may result in the non-functioning of ag85 C. The functioning of ag85 C is crucial for the survival of *M. tuberculosis* in host environment[18]. The results of this investigation suggest that Arg38 and Met125 can be potential targets for antituburcular drugs, as allosteric changes in the structure of these two residues may lead to the non-functioning of ag85 C and hence inactivation of the organism. Active site of ag85 C illustrates the binding mode of the substrate and extends the knowledge concerning specific protein/substrate interactions. This is the first step toward designing potent inhibitors to the mycolyltransferase activity of the ag85 enzymes[13].

## References

[1] Gether U (2000). Uncovering molecular mechanisms involved in activation of G protein coupled receptors. Endocr Rev.

[2] Menon ST, Han M, Sakmar TP (2001). Rhodospin: structural basis of molecular physiology. Physiol Rev.

[3] Hedstrom L, Szilagyi L, Rutter WJ (1993). Converting trypsin to chymotrypsin: the role of surface loops. Science.

[4] Hedstrom L (1996). Trypsin: a case study in the structural determinants of enzyme specificity. Biol Chem.

[5] Patten PA, Gray NS, Yang PL, Marks CB, Wedemayer GY, Jay Boniface J (1996). The immunological evolution of catalysis. Science.

[6] Pertuz MF (1970). Stereochemistry of cooperative effects in haemoglobin. Nature.

[7] Ponting CP, Phillips C, Davies KE, Blake DJ (1997). PDZ domains: targeting signaling molecules to sub-membranous sites. Bioessays.

[8] Lockless SW, Ranganathan R (1999). Evolutionarily conserved pathways of energetic connectivity in protein families. Science.

[9] Suel GM, Lockless SW, Wall MA, Ranganathan R (2003). Evolutionarily conserved networks of residues mediate allosteric communication in proteins. Nat Struct Biol.

[10] Halabi N, Rivoire O, Leibler S, Ranganathan R (2009). Protein sectors: Evolutionary units of three-dimensional structure. Cell.

[11] Ronning DR, Klabunde T, Besra GS, Vissa VD, Belisle JT, Sacchettini JC (2000). Crystal structure of the secreted form of antigen 85C reveals potential drug targets for mycobacterial drugs and vaccines. Nat Struct Biol.

[12] Belisle JT, Vissa VD, Sievert T, Takayama K, Brennan PJ, Besra GS (1997). Role of major antigen of Mycobacterium tuberculosis in cell wall biogenesis. Science.

[13] Ronning DR, Vissa V, Belisle JT, Sacchettini JC (2004). Mycobacterium tuberculosis antigen 85A and 85C structures confirm binding orientations and conserved substrate specificity. J Biol Chem.

[14] Finn RD, Mistry J, Tate J, Coggill P, Heger A, Pollington JE (2010). The Pfam protein families database. Nucleic Acids Research Database Issue.

[15] Waterhouse AM, Procter JB, Martin DMA, Clamp M, Barton GJ (2009). Jalview Version 2 - a multiple sequence alignment editor and analysis workbench. Bioinformatics.

[16] Larkin MA, Blackshields G, Brown NP, Chenna R, McGettigan PA, McWilliam H (2007). Clustal W and Clustal X version 2.0. Bioinformatics.

[17] Fodor Anthony A, Aldrich Richard W (2004). Influence of conservation on calculations of amino acid covariance in multiple sequence alignments. Proteins.

[18] Ward Joe H (1963). Hierarchical grouping to optimize an objective function. J Am Stat Assoc.

